# Urinary Excretion of Silicon in Men, Non-pregnant Women, and Pregnant Women: a Cross-sectional Study

**DOI:** 10.1007/s12011-019-01785-5

**Published:** 2019-06-29

**Authors:** Catarina Magnusson, Ravin Jugdaohsingh, Lena Hulthen, Anna Westerlund, Jonathan J. Powell, Maria Ransjö

**Affiliations:** 1 Department of Orthodontics, Institute of Odontology, The Sahlgrenska Academy, University of Gothenburg, PO Box 450, 405 30 Gothenburg, Sweden; 2 Biomineral Research Group, Department of Veterinary Medicine, University of Cambridge, Madingley Road, Cambridge, CB3 0ES UK; 3 Department of Internal Medicine and Clinical Nutrition, Institute of Medicine, The Sahlgrenska Academy, University of Gothenburg, Box 459, 405 30 Gothenburg, Sweden

**Keywords:** Silicon, Pregnancy, Urinary excretion, Trace element, Bone metabolism

## Abstract

Silicon is a trace element found mainly in plant-based food and proposed to be beneficial for bone health. Urinary excretion of Si has been shown to be a surrogate measure of its uptake in the gastrointestinal tract. The objective of this study was to describe and compare the levels of urinary Si excretion, and consequently Si uptake, in Swedish men, non-pregnant women, and pregnant women. No formal assessment of dietary Si intake was carried out in this study. This cross-sectional study included 89 men, 42 non-pregnant women, and 60 pregnant women. The subjects collected urine over a 24-h period and the samples were assayed for total Si using inductively coupled plasma optical emission spectrometry. The excretion levels of creatinine were used to validate the completeness of the urine sample collections. The mean 24-h urinary excretions of Si were 7.8 mg for the cohort of young men, 7.6 mg for the cohort of non-pregnant women, and 12.4 mg for the cohort of pregnant women. Creatinine excretion was similar between pregnant and non-pregnant women (10.4 vs. 10.8 mmol/day) and significantly higher in men (15.4 mmol/day). The pregnant women excreted significantly higher levels of Si than the young men and non-pregnant women, respectively (*p* < 0.05). The higher urinary excretion of Si by pregnant women compared with men and non-pregnant women is a novel finding possibly caused by temporary physiological changes during pregnancy such as increased gastrointestinal uptake of Si, altered bone metabolism, and increased renal excretion of Si.

## Introduction

Bone is a metabolically active tissue, and bone mass is influenced by hormonal status, physical activity, and nutrition [1]. Calcium and vitamin D are essential nutrients for promoting bone health. Additional dietary factors of importance for bone health are macronutrients such as proteins, micronutrients such as vitamins (e.g. vitamin C), minerals (e.g. Mg and P), and various trace elements (e.g. Zn and Cu) [2]. Another nutrient with possible beneficial properties for bone health is the element silicon (Si). Silicon has been detected in animal bone tissues [3, 4] and localised to the active calcification areas [5]. Early Si deprivation studies in chicks and young rats demonstrated impaired skeletal development and growth of the long bones [6, 7]. In vitro studies have demonstrated that Si stimulates proliferation of osteoblasts, has positive effects on collagen synthesis [8], and inhibits osteoclast differentiation and bone resorption [9]. In line with this, in the Framingham Offspring Cohort, a positive association between Si intake and hip bone mineral density was demonstrated in men and pre-menopausal women, although not in post-menopausal women [10]. Taken together, these studies suggest that Si has positive effects on bone formation and skeletal development.

Si is abundant in the environment and a common dietary component. Whilst there are many Si compounds, the primary specie that humans can assimilate is the soluble orthosilicic acid (OSA), [Si(OH)_4_] [11, 12]. The dietary intake of Si in Western populations is 20–50 mg/day (0.7–1.8 mmol/day) [13–15]. The highest levels of Si are found in cereal-based products, whereas fruits and vegetables have highly variable levels [16] and animal-derived products are low in Si [13]. Whilst the Si content of tap water varies, depending on the surrounding geology, it contributes considerably to daily Si intake due to the large volumes consumed [16]. Alcoholic beverages have moderate to high concentrations of Si [13, 16]; beer provides substantial amounts of bioavailable Si as it is brewed from malted barley [17, 18]. However, foods that have high levels of Si are not necessarily good sources because the absorption of the Si species present in the food depends on how readily they are degraded to OSA in the gastrointestinal tract [19]. The absorption of OSA is reflected by a rapid and marked increase in the serum level of Si following ingestion [12]. Since the kidneys serve as the main route of excretion of Si and the majority of the absorbed Si is readily excreted, urinary levels of Si can be used as a surrogate marker of Si uptake [12, 20]. Fifty to sixty percent of the ingested Si is estimated to be absorbed and excreted in urine, whilst the remainder is not absorbed and found in the faecal excretion [21]. As few studies have investigated 24 h urinary levels of Si and no previous study has reported on Si excretion levels during pregnancy, the aim of the present study was to investigate Si excretion levels in 24-h urine samples collected from Swedish cohorts of young men, non-pregnant women, and pregnant women, to examine gender- and pregnancy-related differences.

## Subjects and Methods

### Subjects

The subjects in the present study were originally participants in the three different epidemiological studies.

#### Cohort I

Data and urine samples from 89 young men, who were originally participants in the Gothenburg Osteoporosis and Obesity Determinants (GOOD) study [22], were used in the present study. The GOOD study was conducted to identify environmental and genetic factors involved in bone and fat mass regulation among young Swedish men. Subjects that met the inclusion criterion of > 18 and < 20 years of age were randomly selected from a national population register and asked to participate. No additional exclusion criteria were set. In total, 1068 men from the greater area of Gothenburg were included in the GOOD study and data were collected in 2005. A representative cross section of subjects from the original study was selected randomly (every tenth subject) to determine the dietary intake and renal excretion levels of Na and K among young Swedish men [23], and these were the subjects included in the present study. In addition to recorded information about their dietary intake by food frequency questionnaire, the subjects also collected urine over a 24-h period.

#### Cohort II

A sub-group of 42 women, formerly participants in a study describing obesogenic trends in two generations of Swedish women [24], were used in the present study. In the original study, 1270 women aged 38 or 50 years were selected based on their date of birth from a population register and were examined either in 1968/1969 or 2004/2005. Data from the last subjects included in to the group and examined in 2004/2005, comprised 26 women aged 50 years and 16 women aged 38 years, were used in the present study. This sub-group of 42 women was comparable with the original group examined. Weekday dietary habits were recorded as 24-h recalls and urine was collected over a 24-h period.

#### Cohort III

Data and urine samples from 60 pregnant women, originally participants in a cross-sectional study (unpublished) completed in 2006 at Gothenburg University, were also analysed in the present study. The aim of the original study was to determine the iodine status and thyroid functions of pregnant women. The inclusion criteria were gestation age between week 8 and week 15 and no pregnancy or lactation in the previous 18 months. No other exclusion criteria were applied. Anthropometric measurements and whole blood and 24-h urine collections were conducted. The consumption of foods with high levels of iodine, such as fish and dairy products, was collected by a food frequency questionnaire and the intake of supplements was also recorded.

### Urine Collections and Analyses

Urine collection was carried out in the same way for all three cohorts. The 24-h collection period started after the first void urine excretion on day 1 and finished after the first void urine excretion on day 2. Urine was collected in acid-washed plastic containers. The total excretion volume was determined, and a 10-ml aliquot was stored at − 20 °C until analysis.

Incomplete 24-h urine collections were identified in all three cohorts based on their urinary creatinine values [25]. Creatinine was measured according to the method routinely used at the accredited Laboratory of Clinical Chemistry at Sahlgrenska University Hospital, Gothenburg, Sweden. Samples with creatinine values outside the normal reference range for men, 8.8–17.6 mmol/day, and for women, 7.0–15.8 mmol/day, were excluded.

Total Si analysis was conducted by the Biomineral Research Group (Cambridge, UK). Frozen urine samples were defrosted at 4 °C. After thorough mixing, a 2-ml aliquot of each sample was diluted with 4 ml of 0.7% nitric acid diluent, prepared by adding 5 ml of high-purity HNO_3_ (69% p.a. plus; Sigma-Aldrich Chemical Co., Gillingham, UK) to a total of 495 ml of ultra-high-purity water (18 MΩ cm). Total Si analysis was carried out on an inductively coupled plasma optical emission spectrometer (ICP-OES, Jobin Yvon 2000-2, Instrument SA, Longjumeau, France), equipped with a concentric nebuliser and cyclonic spray chamber. Sample flow rate was 1 ml/min. Peak profiles were used as previously described [26], with a window size of 0.08 nm (0.04 nm either side of the peak) with 21 increments per profile, and an integration time of 0.5 s per increment. The 251.611-nm Si analytical line was used. Samples were analysed together with sample blanks (i.e. diluent alone) and with pooled sample-based standards (0–40 ppm Si). Pooled sample-based standards were prepared by pooling 1 ml of each of the diluted urine sample and spiking 5–10-ml aliquots of this pooled sample with Si from a stock Si ICP standard solution (1000 ppm Si; Merck Ltd, Poole, UK). Note that ICP-OES quantifies the total amount of elemental Si and gives no information about its chemical composition/speciation.

### Calculations and Statistical Analysis

Due to missing total urine volume values (*n* = 3), creatinine values lying outside the normal range (*n* = 2), and extremely low urinary Si output (*n* = 1), the final cohorts consisted of 86 young men, 40 non-pregnant women, and 59 pregnant women. Body mass index (BMI) was calculated as body weight (kg) divided by the height squared (m^2^). Data were analysed and graphs were made using the PRISM 7 software (GraphPad Software Inc., San Diego, CA, USA). Normal distribution of the data was tested with the Shapiro-Wilk normality test. Confidence interval was set to 95%. Data that passed the normality test were compared with one-way ANOVA followed by Tukey’s multiple comparison test. Non-parametric data were compared with Kruskal-Wallis one-way ANOVA on ranks followed by Dunn’s multiple comparison test. Differences in subject characteristics and urinary Si excretion values between the sub-groups of non-pregnant women of different ages were tested with the Mann-Whitney *U* test. Comparison of the urinary Si levels between the sub-groups of pregnant women taking supplements and those not taking supplements was tested with an unpaired *t* test. Association between BMI and Si excretion was tested with Pearson’s correlation coefficient.

## Results

### Subjects’ Characteristics

The characteristics of the three cohorts are listed in Table 1. The group of men was significantly younger, taller, and heavier than the two groups of women. No difference in the BMI was found between the three groups. There was no significant difference in characteristics between the sub-groups of non-pregnant women aged 38 years and 50 years.

**Table 1:** Characteristics of the study population sub-groups

	Young men			Non-pregnant women									Pregnant women		
	All (a) *N* = 86			All (b) *N* = 40			38-year-olds (c) *N* = 15			50-year-olds (d) *N* = 25			All (e) *N* = 59		
	Mean	SD	Range	Mean	SD	Range	Mean	SD	Range	Mean	SD	Range	Mean	SD	Range
Age (years)	18.7^b,c,d,e^	0.5	18–20	45.5^a,e^	5.9	38–50	38^a^	–	–	50^a,e^	–	–	30.8^a,b,d^	5.4	21–46
Height (m)	1.81^b,c,d,e^	0.06	1.67–1.95	1.65^a^	0.07	1.49–1.81	1.64^a^	0.06	1.49–1.77	1.66^a^	0.07	1.56–1.81	1.67^a^	0.07	1.50–1.79
Weight (kg)	73.6^b,d,e^	11.9	53.1–116.5	65.1^a^	10.5	47.6–99.2	65.5	10.7	47.6–86.4	64.8^a^	10.6	53.6–99.2	67.7^a^	12.2	40.0–109.0
BMI (kg/m^2^)	22.5	3.3	16.8–31.7	23.9	3.5	18.3–34.5	24.3	3.4	18.4–31.4	23.6	3.6	18.3–34.5	24.4	4.3	17.8–38.6
Gestation age (weeks)													11.1	1.2	9–14

Significantly different, *p* < 0.05, compared with ^a^young men (all), ^b^non-pregnant women (all), ^c^non-pregnant women aged 38 years, ^d^non-pregnant women aged 50 years, and ^e^pregnant women (all)

### Urinary Excretion of Si

Total urine volume was significantly lower for men compared with non-pregnant women, but not compared with pregnant women. Whereas no significant difference was found in total urine volume between the groups of women (Table 2). The concentration of Si in urine were significantly different between all three cohorts, with the lowest Si concentration measured in non-pregnant women and the highest in pregnant women (Table 2). As pregnant women were younger than non-pregnant women, we subdivided the non-pregnant women into two groups. However, no significant differences in urine volume or urinary Si concentration, and therefore no significant difference in the total 24-h urinary Si excretion, were found between the sub-groups of non-pregnant women aged 38 years and 50 years (Table 2). Creatinine excretion was similar between pregnant and non-pregnant women (10.4 vs. 10.8 mmol) and significantly higher in men (15.4 mmol; Table 2).

**Table 2:** Urinary profiles of the study population sub-groups

	Young men			Non-pregnant women									Pregnant women		
	All (a) *N* = 86			All (b) *N* = 40			38-year-olds (c) *N* = 15			50-year-olds (d) *N* = 25			All (e) *N* = 59		
	Mean	SD	Range	Mean	SD	Range	Mean	SD	Range	Mean	SD	Range	Mean	SD	Range
Total 24-h urinary creatinine (mmol)	15.4^b,c,d,e^	3.6	6.2–25.5	10.4^a^	2.5	6.3–20.4	11.2^a^	3.4	6.7–20.4	9.9^a^	1.8	6.3–12.8	10.8^a^	2.2	6.7–17.1
Total 24-h urine volume (l)	1.5^b,d^	0.7	0.4–4.1	2.1^a^	0.8	1.1–5.3	1.7	0.7	1.1–3.8	2.3^a^	0.9	1.3–5.3	1.8	0.7	0.5–3.6
24-h urinary Si concentration (mg/l)	5.9^b,d,e^	3.1	1.6–19.1	4.0^a,e^	1.7	1.1–8.7	4.4^e^	1.3	2.4–7.0	3.8^a,e^	1.8	1.1–8.7	7.5^a,b,c,d^	2.9	2.7–16.1
Total 24-h urinary Si excretion (mg)	7.8^e^	3.0	2.4–17.8	7.6^e^	2.9	2.5–15.8	7.2^e^	2.3	3.8–11.1	7.9^e^	3.2	2.5–15.8	12.4^a,b,c,d^	4.7	4.7–24.5

Significantly different, *p* < 0.05, compared with ^a^young men (all), ^b^non-pregnant women (all), ^c^non-pregnant women aged 38 years, ^d^non-pregnant women aged 50 years, and ^e^pregnant women (all)

**Fig. 1:**
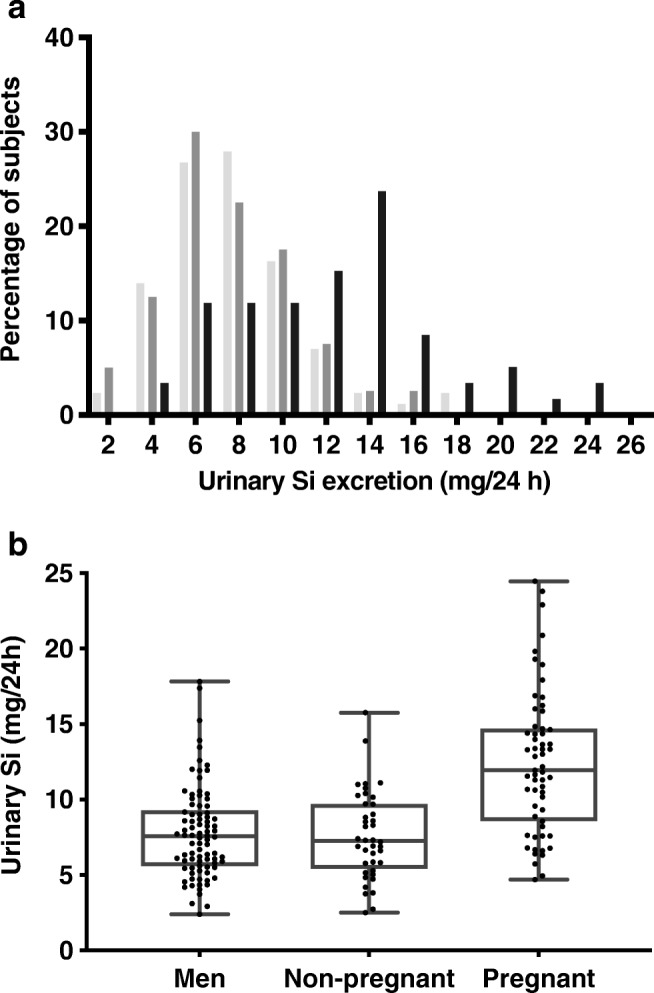
**a** Distribution of relative frequencies of subjects with regard to 24-h urinary Si excretion levels. The young men are represented by light grey bars , non-pregnant women by dark grey bars , and pregnant women by black bars . **b** Distribution of individual urine excretion levels of Si (mg/24 h) within each cohort. Each dot represents an individual subject, the whiskers shows the full data range (min to max), and the box shows the inter-quartile range

With regard to the 24-h urinary excretion of Si, the non-pregnant women and men exhibited a similar distribution pattern (Fig. 1a). However, for the pregnant women, the distribution was shifted towards higher Si levels, as compared with the other two groups (Fig. 1a). The inter-quartile ranges for 24-h Si excretion were as follows: 5.6–9.3 mg for the group of young men; 5.4–9.7 mg for the non-pregnant women; and 8.6–14.7 mg for the pregnant women (Fig. 1b). Mean (± SD) 24-h urinary Si excretion levels were as follows: 7.8 (± 3.0) mg for the cohort of young men; 7.6 (± 2.9) mg for the non-pregnant women; and 12.4 (± 4.7) mg for the cohort of pregnant women, significantly higher (*p* < 0.05) compared with young men and non-pregnant women (Table 2).

**Fig. 2:**
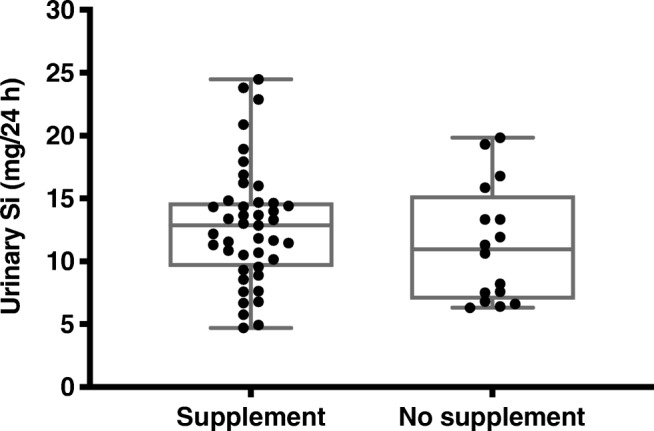
Distribution of individual excretion levels of Si in urine samples from pregnant women taking dietary supplements (*n* = 43) and pregnant women not taking dietary supplements (*n* = 16). Each dot represents an individual subject, the whiskers shows the full data range (min to max), and the box shows the inter-quartile range

Seventy-two percent of the pregnant women (*n* = 43) complemented their diet with supplements on a daily basis. The most common of which was multivitamins, followed by iron and omega-3. The majority of these products did not contain any Si, although in some cases Si was present in negligible amounts as an additive that was needed during formulation. However, there were no significant differences in the urinary Si levels between the pregnant women taking supplements and those not taking supplements, with mean (± SD) of 12.8 (± 4.7) vs. 11.4 (± 4.7) mg/24 h, respectively (Fig. 2).

## Discussion

Urinary levels of Si have never before been reported in pregnant women and the results of the present study revealed significantly higher 24-h urinary excretion of Si in pregnant women, compared with non-pregnant women and young men. The increased urinary Si output in the group of pregnant women was not due to an increase in urine volume but rather to a higher concentration of Si in the urine. This may be due to physiological changes during pregnancy such as increased uptake of Si in the gastrointestinal tract, altered renal excretion of Si, or an increase in bone turnover resulting in an increase in the release of Si from bone and other connective tissues. We also cannot rule out an excessive intake of Si-rich foods as no formal assessment of dietary Si intake was carried out in this study.

Several studies have investigated urinary excretion of Si in relation to different diets, dietary supplementation, and absorption of OSA [17, 21, 27]. Many of the previous studies report Si levels in urine collected over a shorter period than 24 h and therefore, data from these studies cannot be used for comparison. However, the Si levels in the present study agree with the Si concentrations reported in unaffected 24-h urine collections from healthy men and non-pregnant women in a previously conducted study [28]. A study carried out on uraemic patients showed significantly lower 24-h urinary excretion of Si compared with all three cohorts in the present study, confirming that an adequate glomerular filtration rate is a prerequisite for normal urinary Si excretion levels [20]. However, the cohort of pregnant women in the present study had significantly higher total 24-h urinary Si levels compared with healthy men and non-pregnant women presented in the reports of Dobbie and Smith [20, 28]. Another earlier study in healthy individuals reported significantly higher (33.1 mg) 24-h urinary Si excretion levels than those in the present study [29]. The reason for the discrepancy in Si excretion levels is unknown but the analysis of Si is challenging due to the high risk of contamination, as Si is ubiquitous in the environment.

The mechanisms underlying the elevated levels of urinary Si excretion in pregnant women are unknown. A possible explanation for the increased urinary output of Si in the pregnant women is higher Si intake. Our study cannot comprehensively address this question, as the collection of dietary intake data in the pregnant women was selective and focused on foods with high levels of iodine. A limitation of the study is therefore that a comparison of dietary Si intake between the groups cannot be performed. However, a general estimation of the food items recorded in the food frequency questionnaire for the pregnant women shows no distinguishable differences in habitual food intake compared with the other cohorts. The BMI of the pregnant women is not significantly different from that of the other two groups, nor above the limit for overweight which otherwise could be interpreted as deviating dietary habits compared with the other groups. No correlation between BMI and urinary Si excretion was found in the group of pregnant women. Furthermore, epidemiological studies have estimated a significantly higher intake of Si in men than in women [13, 14]. In both studies, beer consumption was the main contributor to the high Si intake in men [13, 14]. Although beer may account for a substantial proportion of daily Si intake and has a high absorption rate, beer consumption varies greatly between individuals and none of the pregnant women in the present study consumed beer on a daily basis. The difference in urinary excretion levels of Si within the groups in the present study probably reflects differences in Si intake but this is unlikely to be the cause for the higher Si excretion values observed for the whole group of pregnant women. Taken together, it is unlikely that these women, in an early stage of pregnancy, would have increased or changed their food intake pattern to such an extent that it would be reflected in the significantly higher levels of Si excretion. Another possible reason for the increased urinary levels of Si in the pregnant women is nutritional supplementation. However, none of the consumed supplements had Si as the active ingredient, and the majority of these products contained negligible amounts of Si (as an additive) in a non-bioavailable form. In addition, statistical analysis reveals no significant difference in urinary excretion levels of Si between pregnant women taking supplements and those not taking supplements.

Pregnancy poses many physiological changes in terms of meeting the demands of the growing foetus. During pregnancy, the placenta actively transports bone-essential elements, primarily calcium (Ca) and phosphorus, to the foetus in order to ensure sufficient mineral concentrations for the developing skeleton [30]. The increased need for Ca is met not only by enhanced gastrointestinal absorption [31] but also possibly by a modified bone status in the mother [30]. It has been suggested that maternal bone mass decreases in the early stages of pregnancy due to an initial phase of bone remodelling [30]. Furthermore, higher urinary excretion of Ca is seen in early pregnancy and an increased glomerular filtration rate is also a possible influencing factor behind the elevated excretion levels [31]. As Si has been proposed to be important for the normal development of bone [6, 7, 32], it is possible that Si is regulated by enhanced absorption and bone metabolism similarly to Ca during pregnancy.

Fasting serum levels of Si could provide further understanding of a potentially altered Si metabolism in pregnant women, but unfortunately the studies in which serum Si levels have been assessed during pregnancy show contradictory results [33, 34]. In one study, significantly lower serum Si levels were reported in pregnant women, as compared with non-pregnant women and the suggested reason for this is that Si is re-distributed from the mother to the growing child for connective tissue development [33]. The lower serum levels of Si in the mothers could also be explained by an expanded plasma volume and consequent haemodilution. Interestingly, the same study also showed significantly increased serum levels of Si in infants (< 1 year of age) compared with adolescents and adults [33]. A more recent study found no significant differences in the serum Si levels between pregnant and non-pregnant women, but reported higher Si levels in the cord blood of new-borns compared with their mothers at birth [34].

## Conclusion

This is the first study to investigate urinary Si excretion in pregnant women and we report that pregnant women have significantly higher 24-h urinary Si excretion levels, compared with the non-pregnant women and men investigated in the current study and compared with previous studies of healthy men and women. We report that this does not appear to be due to altered renal function (urine volume or creatinine excretion). However, as dietary intake was not assessed, we cannot dismiss marked differences in Si intake, although this seems unlikely. A more likely reason for the higher urinary Si excretion in pregnant women is temporary physiological changes such as increased gastrointestinal absorption of Si, altered bone metabolism, and renal excretion of Si, and this deserves further investigation in future studies.
